# Some Account of the African Remittent Fever

**Published:** 1843-09-30

**Authors:** 


*Some Account of the African Remittant Fever which occurred on Board of Her Majesty's Steam Ship Wilberforce, on the River Niger, and whilst engaged on Service on the Western Coast of Africa; comprising an Inquiry into the Causes of Disease in Tropical Climates.* By Morris Pritchett, M. D.

In the 18th No. of the present volume of this Journal, we published a short communication from Dr. J. L. Day, Colonial Physician at Monrovia, on the subject of the cause of the dreaded fever of the African coast. We have before us two highly interesting works, emanating from the late far famed and disastrous Niger expedition, which embody so much that is of the highest interest, that we propose to occupy our readers' attention for a little while, with some important details derived from them. In 1840 three small iron steam-ships, the Albert, London, and Wilberforce, built for river navigation, were fitted out by the British Government, chiefly to contribute to the extinction of the slave trade. The officers were well acquainted with the coast, and a large part of the crew consisted of blacks. The vessels were ventilated by the apparatus of Dr. Reid, by means of which the external atmosphere passing through an iron chamber on the upper deck, could be submitted to the influence of chemical agents. The needles were constructed under the eye of Professor Lloyd of Dublin, so that the influence of the iron was counteracted. In the month of June the vessels reached Sierra Leone, and there completed their crews, from the native Kroomen. They then entered the river, passed the Delta, and proceeded as far as Iddah, the entrance to the valley of the Niger. No case of fever occurred until the 4th of September, when it broke out almost simultaneously in the whole expedition, and did not abate until it was completely paralyzed. Out of the whole expedition there were only fifteen whites who were not attacked with fever in the Niger. With regard to the susceptibility of the negroes to the disease, there seems to be some difference of opinion between our authors. Dr. Pritchett states that those first attacked were men of colour, who had been selected for the business of watering and wooding, and intimates that the disease was of a severe character with them. Dr. McWilliam states, however, that out of one hundred and fifty-eight blacks, eleven only were affected, and these had been for some years out of their respective countries; that the disease was comparatively mild, and that in no instance did it prove fatal. Both agree that a temporary residence in a northern climate renders the negro less liable to resist the causes of disease in hot climates than the white man.

The nature and cause of the fever is fully and ably treated of by Dr. Pritchett. He discards the idea of malaria entirely, asserting that the atmosphere is identical in the wilds of Africa, or the crowded streets of London; at the level of the sea, and at an elevation of sixteen or seventeen thousand feet above the earth's surface. He asserts, amongst other arguments against the doctrine of malaria, that in South America, where you have vegetable matter in a state of decomposition under the influence of a high temperature, there is singular immunity from endemial disease. Among other theories he mentions the latest one—that of Professor Daniell, lately alluded to in this Journal. Having in April, 1840, examined some water from the rivers of the Western Coast of Africa, he detected sulphuretted gas, and immediately ascribed the disastrous epidemics to the influence of this agent. Dr. Pritchett tells us that as soon as they reached the coast of Africa, and on their way up and down the Niger, a rigid examination of the waters was made, the tests being sulphate of copper, nitrate of silver, solution of arsenic, ferrocyanate of potass, carbonate of lead, tartrate of antimony, and the terchloride of gold, and in no instance did they detect any trace of the gas in question. The sulphurretted hydrogen found by Professor Daniell arose from the decomposition of the water during its journey from Africa. Dr. Pritchett remarks that it is not necessary to journey to the mouths of the Niger and Gambia for this gas, as Thames water, kept long enough, will furnish it in abundance. But did it exist in the water this would be no evidence of its deleterious qualities. In the Wilberforce, sulphuretted hydrogen was several times freely evolved from the bursting of jars containing preserved meat; but on no occasion did any evil consequences ensue. The lecture room of the chemist would, were this the case, be often a fruitful source of disease, as well as the most healthful portion of this country, the neighbourhood of the Virginia Springs, together with other sulphur localities of the same nature, both in this country and abroad.

The climate of the Western Coast of Africa is very humid; this moist condition of the air is sometimes abated by the condensation of the vapour into rain, and then respiration is freer and the system feels less oppressed. Dr. P. thinks that remittent fever is a consequence of an effort of nature to restore the equilibrium of the various constituents of the frame, which atmospheric changes had deranged. He says that where the atmosphere between decks was dry, the crews suffered but little. Dr. P. has an original theory on the effect of electricity in the production of river fever, which we have not room to state here. From what he says humidity, with the depressing passions, together with solar influence, are no doubt the chief exciting causes.

To ward off the attacks of the African remittent fever, Dr. Pritchett lays much stress on not overfatiguing the men; in keeping up their spirits; in guarding them against too much exposure to the sun, or to the rain; and on nutritious food. The influence of humidity in producing the disease being recognised, its removal, cannot, he thinks, be too strongly insisted on. Stoves, placed in different parts of the ship, are the best means to keep her dry. The surface of the body should be carefully protected against the damp, chilling air. Our author prefers silk to flannel, from its more perfect non-conducting properties.

Dr. McWilliam has given a somewhat extended account of the invasion and symptoms of the disease. Its approach was often insidious. At first it was intermittent in its type, and then became remittent. Although there was rarely at the commencement a decided chill, the patients generally complained of chilliness. In the hot stage intense dyspnœa was common; but cephalalgia was the most prominent symptom. A subsidence of febrile action generally occurred at the end of three or four hours, and diaphoresis ensued. A disagreeable odour of the perspiration, particularly in the fatal cases, was noticed. The sweating continued from six to twelve hours, when the accession returned. Accessions did not observe any law of periodicity; the evening was perhaps the most common time. In a few instances the remissions were as complete as those in ague; but these were exceptions. The influence of critical days was not apparent. If there were no material improvement beyond the eighth or ninth day the prognosis was gloomy. On the third day the symptoms were aggravated, and at this time Dr. Pritchett states that on feeling the pulse, a hot uneasy sensation remained in the fingers, which could only be removed on washing. The most prominent contingent symptoms were delirium, yellowness of the skin, and convulsions. Petechiæ and sudamina were not observed in any case. In two fatal cases there were lived blotches on the feet and hands, extending to the abdomen and chest. Yellowness of the skin appeared in nineteen cases, thirteen of which were fatal. The average appearance of this symptom was the ninth day. The post mortem examinations were not very scrutinising. The following are the results of nine examinations.

"*Head.*—In two cases where the head was examined, softening was found in the corpus callosum and walls of the ventricles. In one case there was a small quantity of serous fluid in the base of the brain, and an unusual proportion in the ventricles. The dura mater was always sound. The pia mater in one case red and injected. No subarachnoid effusion was observed.

"*Thorax.*—The contents of the thorax were in nearly all cases healthy in appearance. Adhesions between the costal and pulmonary pleuræ were found in one instance, with tubercular deposits in the lungs in the state of induration. In another, a cartilaginous state of the tricuspid valves, with serous effusion in the left pleural sac.

"*Abdomen.*—The peritoneum and its processes, as well as the surface of the intestinal tube, had in general a bilious tinge.

"*The Stomach.*—In several cases the stomach contained from one to five ounces of yellowish-green fluid. The mucous coat was invariably softened, whether this fluid were present or not. In three cases lived patches were variously distributed over the inner surface of the stomach, becoming more distinct when the mucous tunic was scraped off, exhibiting stelliform nuclei in their centres. In two cases, the livid marks were arranged in the form of parallel streaks. These pathological appearances were chiefly in the splenic extremity of the stomach and near the pylorus. In one case there was remarkable venous arborescence on the exterior of the stomach, attended with general engorgement of the portal system. Small points of ulceration were observed in three cases, and slight thickening of the mucous lining in one instance only.

"*Duodenum.*—The lesions observed in the duodenum were of the same nature as those in the stomach, but much less marked. In one case the lower portion of this gut contained a yellowish secretion, of the consistence of mucus.

"*The Jejunum* was free from disease, and likewise the ileum, until within three feet of its lower end, where were observed softening of the mucous lining generally and lived spots. A series of small ulcerations were seen in four cases. In one, the membrane was thickened, rough, and the ulcerations had nearly perforated the bowel; this case proved fatal by terminating in dysentery. The agminated glands of Peyer were distinct and enlarged in three cases.

"*Colon.*—The colon was usually nearly empty. On these occasions a dark, bilious, pultaceous matter was found in this portion of the tube, but in small quantity only; it was viscid and tenacious, adhering to the mucous tunic: where lividity or ulcerated points were found at the lower end of the ileum, the same lesions were seen to exist on the arch of the colon. Softening of the mucous coat was remarkable in three cases. In that of the case of dysentery already mentioned, there was softening of the tunic where it was not ulcerated, and induration and elevation round the edges of the ulcerated patches.

"*Liver.*—The liver was congested in one instance; larger than usual in two cases. It was anemious in two cases where the patients died early, and on two other occasions when death took place long subsequent to febrile action. In the latter cases this organ was of a pale gray colour, and had a dry appearance on being sliced. This condition was not confined to one lobe.

"*Gall-bladder.*—The gall-bladder was distended with bile of the colour and consistence of tar, in three cases; one of which was fatal on the third, one on the seventh, and the other on the ninth day. In another instance the gall-bladder was nearly filled with bloody bile. The man in this case died suddenly, many weeks after the fever had left him.

"The enlarged condition of Peyer's glands, which is regarded by Chomel and Louis as constant in the typhoid fever of France, occurred in three cases out of eight that were examined. In four cases, the subjects of which, with one exception, died early, slight ulcerations of the gastro-enteric mucous membrane were observed. This fact is worthy of attention, inasmuch as it would seem to imply that the cause of the river fever, in whichever way it is introduced into the system, induces an unhealthy action in mucous surfaces much more rapidly than even the low typhoid fevers of France. Chomel does not consider that ulcerations take place in typhoid fevers earlier than the twentieth day, when there is, also, softening of the mucous membrane around the follicles, or in that part of it which covers them. Louis found the patches of Peyer natural in twenty autopsies, made by himself, of yellow fever cases at Gibraltar, during the epidemic of 1828.

"*Spleen.*—In one case the spleen was enlarged, soft, and breaking down under the fingers; in another enlarged, gorged with blood, but firm. This viscus was not altered from the normal condition in the other cases examined. The *pancreas* was not in any case otherwise than natural. The *kidneys* were mottled and larger than usual on one occasion. The *bladder* was in general collapsed. A case in which bloody urine was voided, was not inspected."

With regard to treatment Dr. Pritchett found that the expectant method was the best.

The question of contagion is quickly disposed of. Dr. McWilliam states that no fact came under his notice affording the slightest evidence that the disease was communicable from one person to another. With regard to the question of one attack of river fever affording protection against a second, general experience seems wholly unfavourable to the opinion that any such immunity exists.

